# Baseline working memory was associated with improvement in psychological quality of life in patients with persistent depressive symptoms: a prospective observational study

**DOI:** 10.3389/fpsyt.2026.1838340

**Published:** 2026-05-21

**Authors:** Yuki Oe, Mariko Kurihara, Tomonari Hayasaka, Izumi Nagashima, Masami Murao, Yasuyuki Matsumoto, Yoshikazu Takaesu, Takashi Tsuboi, Koichiro Watanabe, Hitoshi Sakurai

**Affiliations:** 1 Department of Neuropsychiatry, Kyorin University School of Medicine, Mitaka, Japan; 2 Department of Occupational Therapy, Kyorin University School of Health Sciences, Mitaka, Japan; 3 Department of Neuropsychiatry, Graduate School of Medicine, University of the Ryukyus, Ginowan, Japan

**Keywords:** cognition, disability, persistent depression, quality of life, WAIS-IV, working memory

## Abstract

**Background:**

Cognitive dysfunction is prevalent in patients with depressive symptoms and contributes to impaired quality of life (QOL) and functional outcomes. However, the prognostic significance of specific cognitive domains for long-term outcomes remains unclear in patients with persistent depressive symptoms.

**Methods:**

In this prospective observational study, 119 patients completed the detailed examination program in routine clinical care. Of these, 84 patients with persistent depressive symptoms provided consent for study participation and met the eligibility criteria for inclusion in the present study, including completion of the baseline assessment with the Wechsler Adult Intelligence Scale, Fourth Edition (WAIS-IV). The diagnostically heterogeneous cohort included patients with major depressive disorder/dysthymia as well as bipolar and related disorders. The World Health Organization Quality of Life Instrument, Short Version (WHO-QOL-26) and the World Health Organization Disability Assessment Schedule 2.0 were assessed at baseline, three, and six months; 60 participants provided 3-month follow-up data and 50 provided 6-month follow-up data. Primary hierarchical regression analyses were exploratory and conducted using complete-case data.

**Results:**

At baseline, participants exhibited relatively higher verbal ability and lower processing speed compared to normative data. While no significant group-level improvement in QOL was observed over six months and functioning did not improve overall, the WHODAS 2.0 standardized total score was temporarily higher at 3 months than at baseline. In exploratory analyses of complete cases, higher baseline working memory was significantly associated with greater improvement in the psychological domain of the WHO-QOL-26 at six months (β = 0.40; ΔR² = 0.14; p < 0.01). No other cognitive domains showed such associations.

**Conclusions:**

Working memory was associated with subsequent improvement in psychological well-being and may represent a candidate prognostic marker in patients with persistent depressive symptoms. Given the exploratory nature and modest sample size, these findings require replication in larger, diverse populations.

## 1 Introduction

Depression is a prevalent and serious mental disorder worldwide (1). Although many patients achieve remission after sequential evidence-based treatment for a first depressive episode, relapse remains common within the following year (2, 3). Terms such as treatment-resistant depression (TRD) and difficult-to-treat depression (DTD) refer to specific clinical trajectories that have attracted increasing attention because of their complexity and burden (4–6). Consistent with this, recent consensus work has emphasized the heterogeneity of TRD management and the need for more structured treatment pathways in routine care (7). Nevertheless, many patients encountered in clinical practice experience persistent depressive symptoms that do not fit neatly into these categories (8). This heterogeneous group encompasses major depressive disorder (MDD), bipolar disorder, and subthreshold depression, suggesting a broad clinical spectrum that transcends traditional diagnostic boundaries. Accordingly, in the present study, we use the term “patients with persistent depressive symptoms” to refer to the analytic sample.

Mounting evidence suggests that cognitive dysfunction is a core and enduring feature of depression, contributing to both acute symptom burden and long-term functional impairment. Beyond its role as a symptomatic hallmark, cognitive impairment appears to scale with illness complexity. A meta-analysis of patients with first-episode depression reported that 22 of 32 studies found significant cognitive deficits compared with healthy controls, particularly in processing speed, working memory, and verbal memory (9). Crucially, these deficits tend to worsen with illness chronicity and treatment resistance. A large meta-analysis of 11,882 patients in remission from single and recurrent major depressive episodes across 252 studies demonstrated progressive deterioration in executive function, memory, attention, and overall cognitive status with each subsequent depressive episode (10). Consistently, comparative studies have highlighted more pronounced auditory verbal memory and executive dysfunction in TRD populations relative to non-resistant or first-episode patients (11, 12). Collectively, these findings underscore cognitive status as a potential marker of clinical severity and chronicity.

In major depressive disorder, neurocognitive deficits have also been linked to quality of life and to social and occupational functioning, and longitudinal work has suggested that persistent deficits are associated with greater disability and poorer functional recovery (13, 14). A subsequent systematic review further indicated that deficits in executive functioning, attention, and memory are closely associated with functional issues in depression, including quality of life and social functioning (15). Cognitive domains corresponding to the WAIS-IV index scores may also be relevant to quality of life and functioning in depression, although evidence appears stronger for some domains than for others. Prior reviews suggest that executive, attentional, memory, and processing-speed-related deficits are among the cognitive features most consistently associated with psychosocial outcomes in major depressive disorder (13, 15). In addition, baseline working-memory- and processing-speed-related performance has shown potential relevance in treatment-outcome studies, supporting their consideration as plausible candidate predictors (16). By contrast, evidence for domains more closely corresponding to verbal comprehension and perceptual reasoning is less consistent, although broader language and visuospatial abilities may still be relevant to occupational or general functioning in some settings (17).

Despite this established link between cognitive impairment and illness stage, the specific role of baseline cognition as a prospective predictor of clinical recovery remains contentious. A one-year observational study of 131 patients with TRD reported that treatment responders exhibited lower baseline phonemic fluency than non-responders, an unexpected finding that challenges prior assumptions (18). Such discrepancies suggest that the prognostic value of baseline cognitive function remains elusive. Moreover, little is known about how cognitive characteristics relate to clinical outcomes in patients with persistent depressive symptoms across diverse diagnostic categories.

To address this gap, we conducted a prospective observational study to investigate the association between baseline cognitive function and subsequent clinical outcomes, including quality of life (QOL) and functioning, in patients with persistent depressive symptoms who showed inadequate response to standard treatment.

## 2 Materials and methods

### 2.1 Study design

This prospective observational study was conducted between June 2019 and May 2024 at Kyorin University Hospital, Tokyo, Japan. Participants were selected from individuals enrolled in the Kyorin DTD Detailed Examination Program, a multidisciplinary assessment program for patients with persistent depressive symptoms. This program integrated psychiatric, psychological, and biological evaluations to refine diagnosis and formulate individualized treatment recommendations. Patients were referred to the program due to persistent depressive symptoms in routine care. The study was approved by the School of Medicine Research Ethics Committee of Kyorin University (approval no. R01-004; April 22, 2019). All participants provided written informed consent after receiving a full explanation of the study procedures.

### 2.2 Participants

Eligible participants were identified from the program database based on the following inclusion criteria: (1) subjective report of persistent depressive symptoms; (2) retrospectively assessed insufficient improvement during the current depressive episode despite treatment with at least two antidepressant agents; (3) a Montgomery-Åsberg Depression Rating Scale (MADRS) score ≥7 at baseline; and (4) age between 20 and 75 years. Exclusion criteria were: (1) diagnosis of dementia; (2) severe physical illness; and (3) presence of severe suicidal ideation. For the present study, eligibility criterion (2) was operationalized with reference to the treatment guideline of the Japanese Society of Mood Disorders for major depressive disorder (19). Specifically, an adequate antidepressant trial was defined as treatment with an antidepressant at or above the minimum effective dose approved in Japan for at least 4 weeks. Final diagnoses, including bipolar and related disorders, were established through the multidisciplinary assessment after referral and did not preclude inclusion. At the time of referral to the program, participants presented predominantly with persistent depressive symptoms and were not in a manic episode. Many had initially been managed in routine care as having difficult-to-treat depressive conditions before bipolarity was recognized. Accordingly, this threshold was adopted as an operational study criterion and was not intended as a formal diagnosis-specific definition of treatment resistance across all mood disorders. A total of 119 patients completed the detailed examination program in routine clinical care. Of these, 84 provided consent for study participation and met the eligibility criteria for inclusion in the present study. Among the included participants, 60 provided 3-month follow-up data and 50 provided 6-month follow-up data.

### 2.3 Detailed examination program

The examination program comprised a comprehensive evaluation including: (1) detailed psychiatric interviews conducted by multiple psychiatrists; (2) the Structured Clinical Interview for Diagnostic and Statistical Manual of Mental Disorders, Fourth Edition, Text Revision, Axis I Disorders (SCID-I) administered by a clinical psychologist; (3) cognitive testing using the Wechsler Adult Intelligence Scale, Fourth Edition (WAIS-IV); (4) biochemical and physical examinations; and (5) behavioral observation during occupational therapy sessions. Findings were reviewed in a multidisciplinary conference involving psychiatrists, psychologists, occupational therapists, and pharmacists. Although the structured interview was based on DSM-IV-TR criteria, final clinical diagnoses and treatment planning were made through multidisciplinary case review in routine practice, with DSM-5-based clinical judgment also taken into account when appropriate. For all participants, the multidisciplinary findings were summarized in an individually prepared written report. This report was reviewed with each participant by an experienced psychiatrist as part of routine clinical care and included diagnostic impressions and possible treatment directions. However, it was not delivered as a manualized psychological intervention and did not prescribe or standardize subsequent treatment.

### 2.4 Assessment of cognitive function

Baseline cognitive function was evaluated using the WAIS-IV, yielding a Full-Scale Intelligence Quotient (FSIQ) and four index scores: Verbal Comprehension Index (VCI, language ability), Perceptual Reasoning Index (PRI, visual and nonverbal reasoning), Working Memory Index (WMI, auditory attention and working memory/mental manipulation), and Processing Speed Index (PSI, manual task speed).

### 2.5 Outcome measures

QOL was assessed using the World Health Organization Quality of Life Instrument, Short Version (WHO-QOL-26) (20, 21) at baseline, three, and six months. This 26-item measure assesses four domains—physical, psychological, social relationships, and environment—and two global items (overall quality of life and general health); higher scores indicate better QOL. For descriptive presentation, the four WHO-QOL-26 domain values were reported as raw mean item scores for each domain, whereas Overall QOL and General health were reported as raw single-item scores. Functioning was measured using the World Health Organization Disability Assessment Schedule 2.0 (WHODAS 2.0) (22, 23). The 36-item version was administered at baseline, and the 12-item short version at three and six months. To enhance comparability across assessment points, baseline 12-item equivalent scores were derived from the 36-item version by extracting the 12 items corresponding to the official WHODAS 2.0 short version. These items were scored using the WHO simple scoring method, in which responses were recoded from 0 (“none”) to 4 (“extreme/cannot do”), summed to generate a raw summary score, and then converted to a 0–100 standardized score. Higher scores indicate greater impairment. Employment status and follow-up data for QOL and functioning were collected via mailed questionnaires at three and six months, and outcomes were determined based on the returned responses. To examine the robustness of this derivation, we conducted a sensitivity analysis assessing the association between the derived baseline 12-item equivalent standardized score and the full baseline 36-item standardized score.

### 2.6 Statistical analysis

Participants who returned follow-up questionnaires at both 3 and 6 months were included in the complete-case analyses. Demographic and clinical characteristics were summarized at baseline. Socially active status was defined as being employed and not on leave, enrolled as a student, engaged in household duties, or participating in vocational training. To characterize cognitive performance, one-sample t-tests were conducted on the WAIS-IV FSIQ and index scores with the normative population mean of 100. To evaluate changes in QOL and functioning over time, longitudinal changes in the WHO-QOL-26 and WHODAS 2.0 scores were examined from baseline to three and six months using paired t-tests. For descriptive longitudinal summaries, values at each assessment point were presented using all available observations for each outcome. Changes from baseline to each follow-up were calculated using paired data available for each comparison and corresponding 95% confidence intervals and p-values were obtained from paired t-tests.

To identify predictors of outcome changes, hierarchical multiple regression analyses were conducted. Dependent variables were change scores (i.e., follow-up minus baseline) in the WHO-QOL-26 and WHODAS 2.0 scores. In Step 1, age, sex, and the baseline MADRS scores were entered as covariates using the forced-entry method. In Step 2, the WAIS-IV index scores (i.e., VCI, PRI, WMI, and PSI) were entered using a stepwise procedure to determine their unique contribution to outcomes after adjusting for demographic and clinical factors (i.e., age, sex, and baseline depression severity). Given the heterogeneous sample, the modest sample size, and the absence of clearly established domain-specific cognitive predictors in this population, the regression analyses were treated as exploratory and hypothesis-generating. For descriptive longitudinal summaries, available observations at each assessment point were used. By contrast, the primary hierarchical regression analyses were conducted using complete-case data for the relevant change-score outcomes in order to maintain a consistent analytic sample within each model. The 50 participants who completed the 6-month follow-up constituted the main analytic cohort for the primary regression analyses; however, because of additional variable-level missingness, analyzable sample sizes differed slightly across models. Results of all tested hierarchical regression models, including null findings, are presented in **Supplementary Materials**. In addition, 95% confidence intervals for regression coefficients and collinearity indices were examined. Effect-size-related indices, including ΔR² and standardized regression coefficients, were also reported to aid interpretation. Because these analyses were exploratory and hypothesis-generating, no formal multiplicity adjustment was applied; instead, all tested models, including null findings, are reported in the **Supplementary Tables** to facilitate transparent interpretation of the overall pattern of results. As sensitivity analyses, we repeated the main regression model after additionally adjusting for years of education in Step 1. We also repeated the same model in diagnostically restricted samples to examine whether the main finding was materially influenced by diagnostic heterogeneity, specifically by limiting the analysis to unipolar cases and by excluding cases classified as unspecified “other” diagnoses. Because the bipolar-spectrum and “other” subgroups were small, subgroup-specific regression analyses within those groups were considered exploratory and were not interpreted. All analyses were conducted using IBM SPSS Statistics version 28 (IBM Corp., 2021), with statistical significance set at p < 0.05.

## 3 Results

### 3.1 Baseline demographics

**Figure 1:**
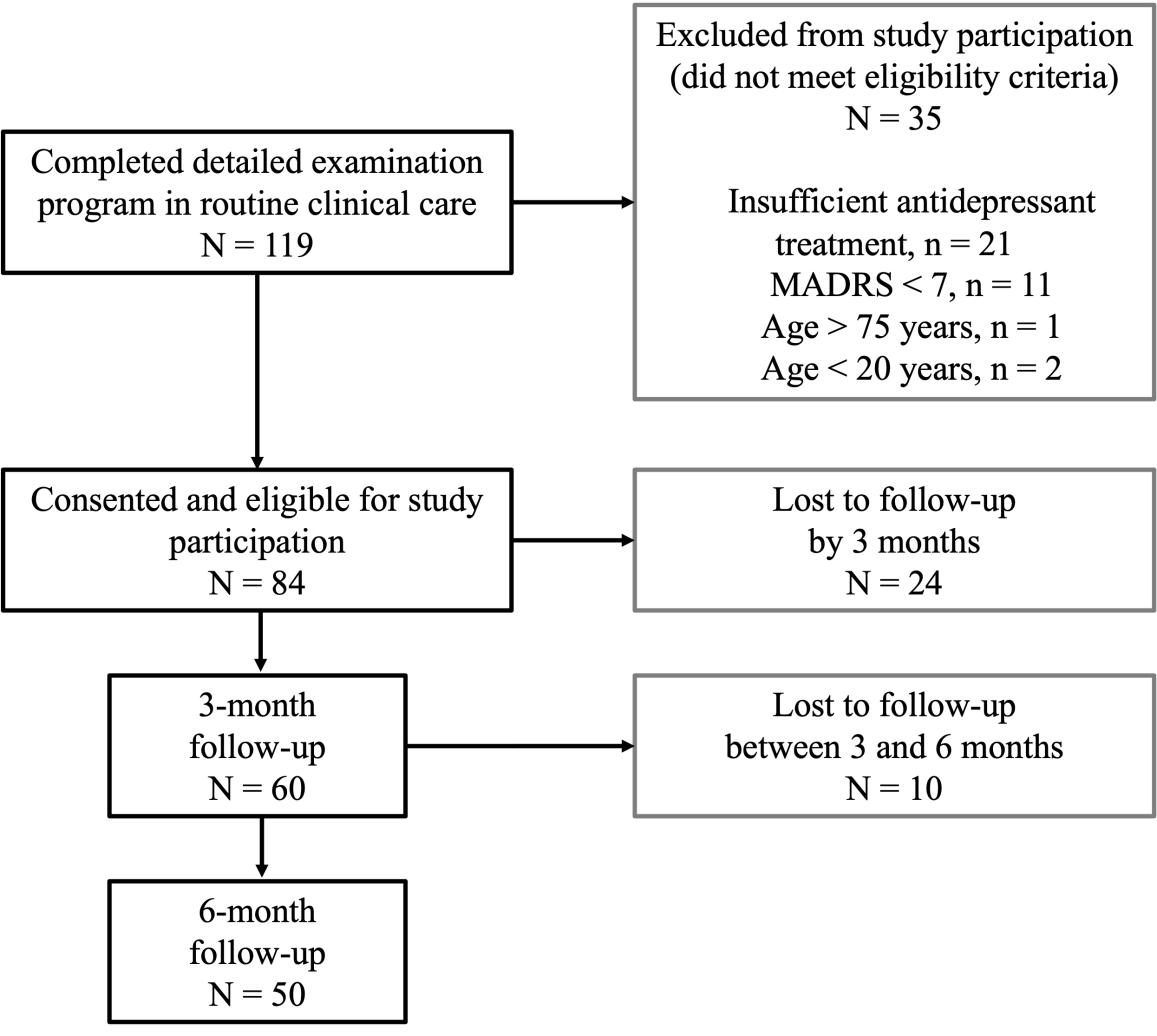
Participant flow. A total of 119 patients completed the detailed examination program in routine clinical care. Of these, 84 provided consent for study participation and met the eligibility criteria for inclusion in the present study dataset, whereas 35 were not included because they did not meet the eligibility criteria and/or met one or more exclusion criteria. Follow-up data were available for 60 participants at 3 months and 50 participants at 6 months. The main analyses were conducted using complete-case data from the 50 participants who completed the 6-month follow-up.

Of the 119 patients who completed the detailed examination program in routine clinical care, 84 were included in the present study dataset after providing consent for study participation and meeting the eligibility criteria. Of these, 60 provided 3-month follow-up data and 50 provided 6-month follow-up data. Descriptive longitudinal summaries were based on all available observations at each time point. The 50 participants who completed the 6-month follow-up constituted the main analytic cohort for the primary regression analyses; however, because of additional variable-level missingness, analyzable sample sizes differed slightly across models. The participant flow is shown in **Figure 1**.

**Table 1** summarizes the baseline characteristics of this analytic cohort (n = 50). The mean age was 38.7 ± 12.0 years, and 27 participants (54.0%) were female. Diagnoses included MDD or dysthymia in 38 participants (76.0%) and bipolar or related disorders in 6 participants (12.0%). Regarding employment status, 25 participants (50.0%) were socially active: 16 (32.0%) were employed, 3 (6.0%) were receiving employment support, 4 (8.0%) were students, and 2 (4.0%) were homemakers. The remaining participants were unemployed (n = 22, 44.0%) or on leave (n = 3, 6.0%). The mean WHO-QOL-26 domain scores at baseline were as follows: physical, 2.5 ± 0.3; psychological, 2.4 ± 0.5; social relationships, 2.8 ± 0.8; and environment, 3.0 ± 0.6. The mean WHODAS 2.0 score was 32.6 ± 14.2. Regarding cognitive function, the mean WAIS-IV FSIQ was 99.7 ± 14.6. Index scores were as follows: 104.6 ± 12.0 for VCI, 101.3 ± 16.6 for PRI, 97.6 ± 15.4 for WMI, and 92.3 ± 14.5 for PSI. Compared with the normative mean of 100, the VCI was significantly higher (t(49) = 2.7, p = 0.01), whereas the PSI was significantly lower (t(49) = −3.7, p < 0.01).

**Table 1:** Baseline characteristics of the analytic sample (n = 50).

Characteristics	Value (n=50)
Age, mean (SD)	38.7 (12.0)
Age of onset, mean (SD)	26.7 (8.8)
Duration of illness, mean (SD)	12.0 (8.0)
Sex	
Male, n (%)	23 (46.0%)
Female, n (%)	27 (54.0%)
Years of education, mean (SD)	14.6 (1.9)
Diagnosis of mood disorders	
Major depressive disorder (Current episode), n (%)	13 (26.0%)
Major depressive disorder (Partial remission), n (%)	19 (38.0%)
Dysthymia, n (%)	6 (12.0%)
Bipolar I disorder, n (%)	2 (4.0%)
Bipolar II disorder, n (%)	3 (6.0%)
Unspecified bipolar and related disorder, n (%)	1 (2.0%)
Others, n (%)	6 (12.0%)
Employment status (last 12 months)	
Employed, n (%)	16 (32.0%)
Vocational rehabilitation, n (%)	3 (6.0%)
Student, n (%)	4 (8.0%)
Homemaker, n (%)	2 (4.0%)
On leave, n (%)	3 (6.0%)
Unemployed, n (%)	22 (44.0%)
MADRS, mean (SD)	20.4 (7.2)
YMRS, mean (SD)	2.1 (2.5)
WHO-QOL-26	
Physical health, mean (SD)	2.5 (0.3)
Psychological, mean (SD)	2.4 (0.5)
Social relationships, mean (SD)	2.8 (0.8)
Environmental health, mean (SD)	3.0 (0.6)
WHODAS 2.0, mean (SD)	32.6 (14.2)
WAIS-IV	
Full Scale-IQ, mean (SD)	99.7 (14.6)
Verbal comprehension index, mean (SD)	104.6 (12.0)
Perceptual reasoning index, mean (SD)	101.3 (16.6)
Working memory index, mean (SD)	97.6 (15.4)
Processing speed index, mean (SD)	92.3 (14.5)

SD, standard deviation; MADRS, Montgomery-Åsberg Depression Rating Scale; YMRS, Young Mania Rating Scale; WHO-QOL-26, World Health Organization Quality of Life Instrument, Short Version; WHODAS 2.0, World Health Organization Disability Assessment Schedule 2.0; WAIS-IV, Wechsler Adult Intelligence Scale, Fourth Edition; IQ, Intelligence Quotient.

WHO-QOL-26 domain values are presented as raw mean item scores. The WHODAS 2.0 value shown here is the baseline derived 12-item equivalent standardized score from the 36-item baseline administration.

Of the 84 participants included in the present dataset, 50 completed the 6-month follow-up and 34 did not. Because descriptive longitudinal summaries used available observations and the primary regression analyses used complete-case data for the relevant outcomes, we examined baseline differences between completers and non-completers to assess potential attrition bias. There was no significant difference between completers and non-completers in sex distribution (χ²(1) = 0.39, p = 0.53) or diagnostic group classification (χ²(2) = 0.13, p = 0.94). Likewise, age, years of education, MADRS score, YMRS score, baseline psychological QOL, baseline WHODAS standardized score, WAIS-IV perceptual reasoning, working memory, and processing speed indices, and illness duration did not differ significantly between groups. However, non-completers had a higher verbal comprehension index than completers (111.1 vs. 104.3, p = 0.02). Detailed results are provided in **Supplementary Table 1**.

At baseline, 45 of 50 participants (90.0%) were receiving at least one psychotropic medication. Antidepressants were the most frequently prescribed class (66.0%), followed by benzodiazepine anxiolytics/hypnotics (52.0%) and antipsychotics (44.0%). Psychotropic polypharmacy was common, with 64.0% receiving two or more psychotropic agents (**Table 2**). Descriptive information on psychiatric and physical comorbidities is also summarized in **Table 3**. Among the 50 participants, 36 (72.0%) had at least one non-mood psychiatric comorbidity. Anxiety-related disorders were the most common (30/50, 60.0%), followed by somatoform-related disorders (16/50, 32.0%), substance-related disorders (6/50, 12.0%), and eating disorders (5/50, 10.0%). In addition, 12 participants (24.0%) had physical comorbidities potentially relevant to mood symptoms, most commonly sleep-related disorders (5/50, 10.0%) and endocrine/metabolic disorders (5/50, 10.0%).

**Table 2:** Baseline psychotropic medications.

Variables	Value (n = 50)
Any psychotropic medication, n (%)	45 (90.0%)
Antidepressants, n (%)	33 (66.0%)
Antipsychotics, n (%)	22 (44.0%)
Mood stabilizers, n (%)	11 (22.0%)
Benzodiazepines, n (%)	26 (52.0%)
Other hypnotics, n (%)	8 (16.0%)
Number of psychotropic medications, median [IQR]	2 [1-4]
Polypharmacy (≥2 psychotropic agents), n (%)	32 (64.0%)

IQR, interquartile range.

**Table 3:** Baseline psychiatric and physical comorbidities.

Comorbidities	N (%)
Any non-mood psychiatric comorbidity	36 (72.0%)
Any anxiety-related disorder	30 (60.0%)
Any somatoform-related disorder	16 (32.0%)
Any substance-related disorder	6 (12.0%)
Any eating disorder	5 (10.0%)
Any physical comorbidity potentially relevant to mood symptoms	12 (24.0%)
Sleep-related disorders	5 (10.0%)
Endocrine/metabolic disorders	5 (10.0%)
Neurological/systemic medical conditions	3 (6.0%)
Gastrointestinal conditions	2 (4.0%)
Gynecological conditions	1 (2.0%)

Categories are not mutually exclusive.

### 3.2 Changes in social activity, QOL, and functioning over time

Socially active status was observed in 51.0% of participants at three months and 55.0% at six months. Descriptive longitudinal data for each WHO-QOL-26 domain, the two global items, and the standardized total WHODAS 2.0 score are presented in **Table 4** using all available observations at each assessment point. Sample sizes varied slightly across outcomes because of missing data. Mean changes from baseline, together with 95% confidence intervals and p-values from paired comparisons, are also shown in **Table 4**. Paired t-tests revealed no statistically significant changes in WHO-QOL-26 scores from baseline to three or six months. The WHODAS 2.0 standardized total score was significantly higher at 3 months than at baseline, indicating greater self-reported disability at that time point, whereas the baseline-to-6-month change was not significant. Although the mean changes in most outcomes did not reach statistical significance, substantial individual variability was observed (e.g., psychological QOL change at six months: mean = 0.10, SD = 0.51, range = −0.83 to 2.00). Paired comparisons showed no significant mean improvement in QOL or functioning at the group level over the 6-month follow-up. The main finding of the present study therefore concerns between-patient variation in change.

**Table 4:** Longitudinal descriptive data and changes from baseline in WHO-QOL-26 and WHODAS 2.0 scores.

Characteristics	BL, mean (SD), n	3-month FU, mean (SD), n	Change from BL to 3-month FU, mean (95% CI), p value	6-month FU, mean (SD), n	Change from BL to 6-month FU, mean (95% CI), p value
WHO-QOL-26					
Physical health	2.42 (0.33), 84	2.47 (0.36), 57	+0.04 (−0.04 to 0.12), p = 0.34	2.52 (0.45), 49	+0.04 (−0.09 to 0.18), p = 0.51
Psychological	2.39 (0.47), 84	2.44 (0.47), 58	+0.05 (−0.06 to 0.16), p = 0.34	2.52 (0.55), 49	+0.10 (−0.04 to 0.25), p = 0.17
Social relationships	2.77 (0.76), 84	2.66 (0.87), 56	−0.10 (−0.32 to 0.12), p = 0.39	2.78 (0.69), 49	+0.01 (−0.21 to 0.24), p = 0.90
Environmental health	3.08 (0.59), 84	2.92 (0.70), 57	−0.08 (−0.24 to 0.08), p = 0.34	3.06 (0.68), 50	+0.06 (−0.13 to 0.25), p = 0.55
Overall QOL	1.96 (0.78), 84	2.05 (0.83), 58	+0.05 (−0.18 to 0.29), p = 0.66	2.26 (0.85), 50	+0.20 (−0.09 to 0.49), p = 0.18
General health	1.56 (0.67), 84	1.64 (0.72), 58	0.00 (−0.25 to 0.25), p = 1.00	1.76 (0.80), 50	+0.08 (−0.17 to 0.33), p = 0.52
WHODAS 2.0					
Standardized total score	46.35 (13.28), 84	50.63 (15.50), 58	+4.60 (0.97 to 8.23), p = 0.01	46.17 (16.74), 50	+0.84 (−3.46 to 5.14), p = 0.70

BL, baseline; FU, follow-up; SD, standard deviation; CI, confidence interval; WHO-QOL-26, World Health Organization Quality of Life Instrument, Short Version; QOL, quality of life; WHODAS 2.0, World Health Organization Disability Assessment Schedule 2.0.

Values at each assessment point are shown using all available observations for each outcome. Because of missing data, sample sizes varied slightly across variables and time points. Changes from baseline were calculated as follow-up minus baseline using paired data available for each comparison. WHO-QOL-26 domain values are presented as raw mean item scores for each domain; Overall QOL and General health are presented as raw single-item scores. Baseline WHODAS 2.0 values represent a derived 12-item equivalent standardized score from the 36-item baseline administration. Higher WHO-QOL-26 scores indicate better quality of life, whereas higher WHODAS 2.0 standardized total scores indicate greater disability.

### 3.3 Impact of cognition on subsequent improvement in QOL and functioning

**Figure 2:**
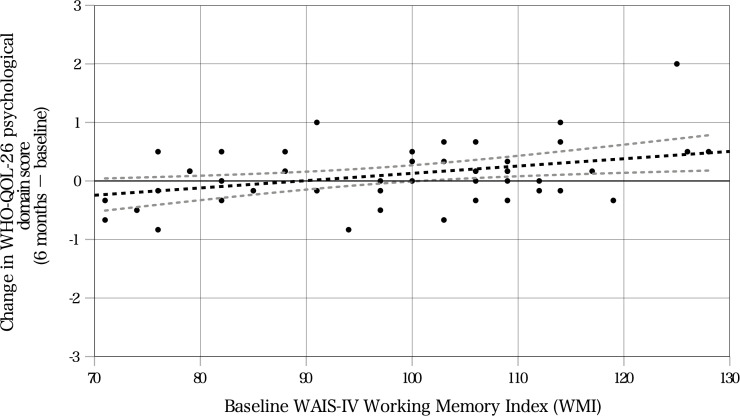
Association between baseline working memory and 6-month improvement in psychological quality of life (analyzable n = 49). Scatter plot illustrating the relationship between baseline Wechsler Adult Intelligence Scale, Fourth Edition Working Memory Index and the change score in the psychological domain of the WHO-QOL-26 (6 months minus baseline). The black dashed line indicates the fitted regression line, and the gray dashed lines indicate the 95% confidence bands. Positive values indicate improvement.

Results of all exploratory hierarchical regression models, including null findings, are presented in **Supplementary Table 2** and S3. Across these exploratory models, only one significant association emerged: higher baseline WMI was associated with greater improvement in the psychological domain of the WHO-QOL-26 at 6 months. In the primary model, adding baseline WMI significantly increased the explained variance (ΔR² = 0.14, F(1, 44) = 7.60, p = 0.01), resulting in a final model R² of 0.19 (β = 0.40, p < 0.01). The regression model for 6-month change in the psychological domain of the WHO-QOL-26 was based on 49 participants with complete data for that specific analysis (**Table 5**). No other WAIS-IV index scores were retained as significant predictors of QOL or disability outcomes. Although the cohort as a whole did not show significant average improvement, substantial individual variability in change scores was observed, supporting the exploratory analysis of predictors of between-patient differences in outcome trajectories. The relationship between baseline WMI and improvement in psychological QOL at 6 months is illustrated in **Figure 2**.

**Table 5:** Predictors of change in psychological domain of QOL at six months.

Variables	Model 1, B (SE)	β	Model 2, B (SE)	β
Age	0.00 (0.01)	0.01	0.00 (0.01)	−0.02
Sex	0.23 (0.16)	0.22	0.22 (0.15)	0.22
MADRS	−0.03 (0.10)	–0.04	0.01 (0.01)	0.09
WAIS-IV Working Memory Index	n.a.	n.a.	0.01 (0.01)	0.40**
R²	0.05		0.19	
ΔR² (Model 2 – Model 1)			0.14	
F change (df1, df2)			7.60 (1, 44)	
p for change			< 0.01	

Dependent variable: change in psychological QOL (WHO-QOL-26 psychological domain; 6 months minus baseline).

Sex coded as 0 = female, 1 = male.

QOL, quality of life; SE, standard error; MADRS, Montgomery-Åsberg Depression Rating Scale; WAIS-IV, Wechsler Adult Intelligence Scale, Fourth Edition; n.a., not available.

** p < 0.01.

The analyzable sample size for this model was n = 49 because one participant had missing data required to calculate the 6-month change score for the psychological WHO-QOL-26 domain.

In sensitivity analyses additionally adjusting for years of education, the association between baseline WMI and improvement in the psychological domain of the WHO-QOL-26 at 6 months remained significant (β = 0.45, ΔR² = 0.16, p = 0.01). Similar results were observed when the analysis was restricted to unipolar cases (β = 0.41, ΔR² = 0.15, p = 0.03) and when cases with unspecified “other” diagnoses were excluded (β = 0.46, ΔR² = 0.17, p = 0.01). The results of these sensitivity analyses are summarized in **Supplementary Table 4**. Because the bipolar-spectrum and “other” subgroups were very small, subgroup-specific regression models within those groups yielded unstable estimates and were not interpreted further.

## 4 Discussion

Few prospective studies have examined the association between baseline cognitive function and subsequent changes in QOL and functioning in patients with persistent depressive symptoms. The present sample exhibited higher verbal ability and lower processing speed compared to normative data. Higher baseline working memory was associated with greater improvement in the psychological domain of QOL at six months. These findings suggest that specific cognitive domains, particularly working memory, may represent candidate prognostic markers for psychological well-being in patients with persistent depressive symptoms and provide a basis for future hypothesis-driven research.

The present sample exhibited a cognitive profile characterized by markedly reduced processing speed in the context of relatively preserved verbal abilities. Similar patterns have been reported in cross-sectional comparisons between TRD/DTD and non-resistant populations, where slowed processing speed often coincides with broader impairments in executive functioning and memory (11, 12). Importantly, a large meta-analysis including 11,882 patients demonstrated that a greater number of prior depressive episodes was associated with slower processing speed even during remission, suggesting that this impairment reflects cumulative illness burden rather than a purely state-dependent effect (10). Consistent with this interpretation, residual symptoms such as reduced energy and concentration difficulties commonly persist after remission (24), and processing speed tasks, which are highly time-constrained, may be particularly sensitive to these residual features. Moreover, reduced processing speed may limit the effective deployment of higher-order cognitive abilities, such that preserved cognitive capacity is not efficiently translated into real-world performance (25). Taken together, these findings suggest that slowed processing speed represents a common and enduring cognitive characteristic in persistent depressive symptoms and may serve as a marker of illness chronicity.

Our longitudinal findings indicate that working memory may provide meaningful information for understanding subsequent changes in psychological well-being. These findings should nevertheless be interpreted cautiously. Only one significant association emerged across multiple exploratory models, and the cohort as a whole did not show significant mean improvement in QOL or functioning over time. The principal finding therefore concerns between-patient variation in change rather than overall clinical recovery. In addition, the key outcome was the psychological domain of a self-reported QOL measure; thus, the observed association links performance-based cognitive assessment with a subjective patient-reported outcome. One possible interpretation is that better working memory may facilitate the retention and integration of treatment-related information, including individualized feedback provided during the multidisciplinary assessment process. However, this mechanistic explanation remains hypothetical and was not directly tested. The routine individualized feedback process embedded in the assessment program may also have functioned as a psychoeducational element, although this possibility was not directly examined in the present study. Ongoing pharmacological treatment and the routine individualized feedback process embedded in the assessment program may also have influenced subsequent self-reported outcomes, and their respective contributions could not be disentangled.

It is possible that self-perceived psychological well-being is more sensitive to early change than broader disability measures, which may partly explain why an association was observed for the psychological WHO-QOL-26 domain but not for WHODAS 2.0. This interpretation is consistent with previous evidence suggesting that symptomatic recovery and social or functional recovery do not necessarily proceed in parallel in depression (26, 27). Broader disability may require more sustained behavioral and social change before measurable improvement becomes apparent. One possible explanation for the temporary increase in WHODAS 2.0 scores at 3 months is that the detailed assessment and individualized feedback process may have increased participants’ awareness of their day-to-day difficulties, resulting in higher short-term self-reported disability. However, this interpretation remains speculative and was not directly tested in the present study. In addition, although the derived baseline 12-item equivalent WHODAS score showed a very high correlation with the full baseline 36-item standardized score, the use of different WHODAS versions across time points should also be kept in mind when interpreting disability-related findings.

This discrepancy may reflect domain-specific change, in which a narrower psychological quality-of-life domain may show improvement even when broader disability outcomes remain stable. This interpretation is broadly consistent with recent esketamine research showing that anhedonia, a clinically meaningful psychological dimension, may improve distinctly from overall depressive symptom reduction (28). More broadly, the present findings may also be interpreted in light of emerging work that moves beyond purely symptom-centered models toward more personalized and mechanism-informed understandings of recovery in treatment-resistant depression (29). These findings may also be relevant to considering patient-centered and possibly individualized support strategies, an interpretation that is broadly consistent with recent multidisciplinary models emphasizing that treatment-related support may be shaped by how care is delivered, monitored, and integrated over time (30). Future research could examine whether support strategies designed to reduce working-memory demands, such as visual supports and structured summaries, facilitate patients’ learning and retention of treatment-related information in patients with persistent depressive symptoms (31).

The main finding was broadly consistent across limited sensitivity analyses adjusting for years of education and addressing broad diagnostic composition. However, because the bipolar-spectrum subgroup was small, these analyses do not allow firm conclusions regarding subgroup-specific effects.

Several limitations should be noted. First, the sample was diagnostically heterogeneous, encompassing major depressive disorder, dysthymia, and bipolar and related disorders, which may have obscured diagnosis-specific cognitive patterns. Only 50 of the 84 participants included in the present study dataset provided data at both follow-up time points and were therefore included in the complete-case analyses, raising the possibility of attrition-related selection bias. The relatively small sample size also limits the statistical power of the multivariable analyses and increases the risk of overfitting, particularly in the stepwise regression models. In addition, restricting the analyses to complete cases may have reduced the use of available information, particularly at 3 months, when follow-up data were available for 60 participants. Attrition bias should also be considered when interpreting the findings. Although completers and non-completers did not differ significantly in most baseline demographic and clinical characteristics, non-completers had a higher verbal comprehension index than completers.

Thus, while broad attrition-related bias appeared limited overall, some selective differences cannot be excluded. Although descriptive information on baseline psychotropic treatment has now been added, medications and clinical changes were not systematically tracked during follow-up. Accordingly, ongoing pharmacological treatment may have influenced both baseline cognitive performance and subsequent self-reported outcomes, and its contribution could not be disentangled from that of other clinical factors, including the routine individualized feedback process. Another limitation is that psychiatric and relevant medical comorbidities were common in this sample and may have influenced both baseline cognitive performance and longitudinal quality-of-life outcomes. Although descriptive information on these comorbidities has been added, the present study was not designed to statistically disentangle their individual effects. Accordingly, the association between baseline working memory and subsequent psychological QOL should be interpreted cautiously. The use of different WHODAS 2.0 versions (36-item vs. 12-item) requires cautious interpretation, although the derived baseline 12-item equivalent standardized score showed a very high correlation with the full baseline 36-item standardized score in a sensitivity analysis. Because the two versions are not identical, disability-related findings should still be interpreted cautiously. The use of stepwise selection means the observed association between working memory and psychological QOL improvement should be regarded as exploratory and hypothesis-generating. In addition, because multiple outcomes, time points, and cognitive indices were examined, the possibility of type I error should be acknowledged. The complete-case regression approach may also have reduced the use of available information. A mixed-effects model using all repeated observations would provide a more robust analytic framework for future confirmatory studies of incomplete longitudinal data. Finally, the exclusive use of self-report measures for QOL and functioning introduces the possibility of subjective bias.

In conclusion, this study provides preliminary evidence that baseline working memory may be associated with subsequent improvement in psychological QOL among patients with persistent depressive symptoms. Although these findings are hypothesis-generating, they suggest that cognitive differences may be clinically relevant to understanding heterogeneity in subsequent psychological well-being. These findings require confirmation in larger, better-characterized longitudinal cohorts.

## Data Availability

The datasets generated and/or analyzed during the current study are not publicly available because they contain participant-level clinical information and are subject to ethical and privacy restrictions. Requests to access the datasets should be directed to the corresponding author and will be considered on reasonable request and with appropriate institutional approval.
