# Supplementary material for: Baseline working memory was associated with improvement in psychological quality of life in patients with persistent depressive symptoms: a prospective observational study
Source: Front Psychiatry. 2026 May 21;17:1838340. doi: 10.3389/fpsyt.2026.1838340 (PMC13233697; doi:10.3389/fpsyt.2026.1838340)
Supplement: Supplementary file 1 [file SupplementaryFile1.docx]

Supplementary Material

Supplementary Table S1. Baseline characteristics of completers and non-completers

| Variable | Non-completers (n = 34) | Completers (n = 50) | Statistic | p value |
| --- | --- | --- | --- | --- |
| **Categorical variables** |  |  |  |  |
| Sex |  |  | χ²(1) = 0.4 | 0.53 |
| male, n (%) | 18 (52.9) | 23 (46.0) |  |  |
| female, n (%) | 16 (47.1) | 27 (54.0) |  |  |
| Diagnostic group |  |  | χ²(2) = 0.1 | 0.94 |
| Depressive disorder group, n (%) | 25 (73.5) | 38 (76.0) |  |  |
| Bipolar spectrum group, n (%) | 5 (14.7) | 6 (12.0) |  |  |
| Other psychiatric disorders group, n (%) | 4 (11.8) | 6 (12.0) |  |  |
| **Continuous variables** |  |  |  |  |
| Age, years, mean (SD) | 36.6 (10.3) | 38.7 (12.0) | t(82) = −0.85 | 0.40 |
| Years of education, mean (SD) | 14.8 (2.5) | 14.6 (1.9) | t(82) = 0.39 | 0.70 |
| Illness duration, years, mean (SD) | 8.9 (5.7) | 12 (8.0) | t(82) = −1.96 | 0.05 |
| MADRS, mean (SD) | 19.9 (8.1) | 20.4 (7.2) | t(82) = −0.28 | 0.78 |
| YMRS, mean (SD) | 2.1 (2.7) | 2.1 (2.5) | t(81) = −0.08 | 0.94 |
| Baseline psychological QOL, mean (SD) | 2.3 (0.5) | 2.4 (0.5) | t(82) = −1.14 | 0.26 |
| Baseline WHODAS standardized score, mean (SD) | 47.8 (13.0) | 45.33 (13.5) | t(82) = 0.85 | 0.40 |
| WAIS |  |  |  |  |
| Verbal comprehension index, mean (SD) | 111.1 (13.3) | 104.3 (12.0) | t(82) = 2.43 | 0.02 |
| Perceptual reasoning index, mean (SD) | 105.8 (14.5) | 101.3 (16.6) | t(82) = 1.23 | 0.22 |
| Working memory index, mean (SD) | 101.2 (13.6) | 97.6 (15.4) | t(82) = 1.11 | 0.27 |
| Processing speed index, mean (SD) | 93.5 (15.2) | 92.3 (14.5) | t(82) = 0.38 | 0.71 |

MADRS, Montgomery–Åsberg Depression Rating Scale; YMRS, Young Mania Rating Scale; QOL, quality of life; WHODAS, World Health Organization Disability Assessment Schedule 2.0; WAIS-IV, Wechsler Adult Intelligence Scale, Fourth Edition.

Values are presented as mean (SD) or n (%). Categorical variables were compared using chi-square tests, and continuous variables were compared using independent-samples t-tests. Non-completers were defined as participants who did not complete the 6-month follow-up.

Supplementary Table S2. Exploratory hierarchical regression models for 3-month change in QOL and disability outcomes

| Outcome | N | Step 1 variables | R^2^ | Adj. R^2^ | F (df) | p | Retained WAIS-IV index | ΔR^2^ | F change (df) | p change | B (SE) | β | 95% CI | Max VIF | Note |
| --- | --- | --- | --- | --- | --- | --- | --- | --- | --- | --- | --- | --- | --- | --- | --- |
| Change in physical domain | 50 | Age, sex, MADRS | 0.08 | 0.02 | 1.32 (3, 46) | 0.28 | None | - | - | - | - | - | - | 1.12 | No index retained |
| Change in Psychological domain | 50 | Age, sex, MADRS | 0.03 | −0.03 | 0.51 (3, 46) | 0.68 | None | - | - | - | - | - | - | 1.12 | No index retained |
| Change in social domain | 49 | Age, sex, MADRS | 0.12 | 0.06 | 2.04 (3, 45) | 0.12 | None | - | - | - | - | - | - | 1.12 | No index retained |
| Change in environment domain | 50 | Age, sex, MADRS | 0.32 | 0.28 | 7.21 (3, 46) | <0.01 | None | - | - | - | - | - | - | 1.12 | No index retained |
| Change in Overall QOL | 50 | Age, sex, MADRS | 0.27 | 0.22 | 5.53 (3, 46) | <0.01 | None | - | - | - | - | - | - | 1.12 | No index retained |
| Change in General health | 50 | Age, sex, MADRS | 0.14 | 0.08 | 2.51 (3, 46) | 0.07 | None | - | - | - | - | - | - | 1.12 | No index retained |
| WHODAS 2.0 total change | 50 | Age, sex, MADRS | 0.15 | 0.09 | 2.60 (3, 46) | 0.06 | None | - | - | - | - | - | - | 1.12 | No index retained |

MADRS, Montgomery–Åsberg Depression Rating Scale; WAIS-IV, Wechsler Adult Intelligence Scale–Fourth Edition; QOL, quality of life; WHODAS 2.0, World Health Organization Disability Assessment Schedule 2.0; VIF, variance inflation factor; CI, confidence interval.

Supplementary Table S3. Exploratory hierarchical regression models for 6-month change in QOL and disability outcomes

| Outcome | N | Step 1 variables | R^2^ | Adj. R^2^ | F (df) | p | Retained WAIS-IV index | ΔR^2^ | F change (df) | p change | B (SE) | β | 95% CI | Max VIF | Note |
| --- | --- | --- | --- | --- | --- | --- | --- | --- | --- | --- | --- | --- | --- | --- | --- |
| Change in physical domain | 49 | Age, sex, MADRS | 0.13 | 0.08 | 2.33 (3, 45) | 0.09 | None | - | - | - | - | - | - | 1.13 | No index retained |
| Change in Psychological domain | 49 | Age, sex, MADRS | 0.05 | −0.01 | 0.85 (3, 45) | 0.47 | WMI | 0.14 | 7.60 (1, 44) | 0.01 | 0.01 (0.01) | 0.40 | 0.00 to 0.02 | 1.12 | Only retained index |
| Change in social domain | 49 | Age, sex, MADRS | 0.04 | −0.02 | 0.67 (3, 45) | 0.57 | None | - | - | - | - | - | - | 1.12 | No index retained |
| Change in environment domain | 50 | Age, sex, MADRS | 0.20 | 0.15 | 3.84 (3, 46) | 0.02 | None | - | - | - | - | - | - | 1.12 | No index retained |
| Change in Overall QOL | 50 | Age, sex, MADRS | 0.03 | −0.04 | 0.42 (3, 46) | 0.74 | None | - | - | - | - | - | - | 1.12 | No index retained |
| Change in General health | 50 | Age, sex, MADRS | 0.01 | −0.05 | 0.16 (3, 46) | 0.92 | None | - | - | - | - | - | - | 1.12 | No index retained |
| WHODAS 2.0 total change | 50 | Age, sex, MADRS | 0.06 | −0.01 | 0.88 (3, 46) | 0.46 | None | - | - | - | - | - | - | 1.12 | No index retained |

MADRS, Montgomery–Åsberg Depression Rating Scale; WAIS-IV, Wechsler Adult Intelligence Scale–Fourth Edition; WMI, Working Memory Index; QOL, quality of life; WHODAS 2.0, World Health Organization Disability Assessment Schedule 2.0; VIF, variance inflation factor; CI, confidence interval.

Hierarchical multiple regression analyses were conducted with age, sex, and baseline MADRS score entered in Step 1, followed by stepwise entry of WAIS-IV index scores in Step 2. Step 1 model statistics are shown as R², adjusted R², F, and p. Step 2 statistics are shown only for retained WAIS-IV index scores; a dash indicates that no index was retained. Positive change scores indicate higher follow-up scores relative to baseline.

Supplementary Table S4. Sensitivity analyses for the association between baseline working memory and change in the psychological domain of WHO-QOL-26 at 6 months

| Models | N | Step 1 covariates | R^2^ | Step 2 selected predictor | B | 95% CI | Standardized β | ΔR^2^ | p |
| --- | --- | --- | --- | --- | --- | --- | --- | --- | --- |
| Full sample | 49 | Age, sex, MADRS, years of education | 0.08 | WMI | 0.02 | 0.01 to 0.03 | 0.45 | 0.16 | 0.01 |
| Unipolar cases only | 37 | Age, sex, MADRS, years of education | 0.03 | WMI | 0.02 | 0.00 to 0.03 | 0.41 | 0.15 | 0.03 |
| Excluding unspecified “other” diagnoses | 43 | Age, sex, MADRS, years of education | 0.12 | WMI | 0.02 | 0.01 to 0.03 | 0.46 | 0.17 | 0.01 |

MADRS, Montgomery–Åsberg Depression Rating Scale; WMI, Working Memory Index; WHO-QOL-26, World Health Organization Quality of Life Instrument, Short Version.

In all models, age, sex, MADRS score, and years of education were entered in Step 1. WAIS-IV index scores were entered in Step 2 using a stepwise method. Regression analyses limited to the bipolar-spectrum subgroup and the unspecified “other” subgroup were not interpreted because the sample sizes were too small to provide stable estimates.
